# Efficacy of acupuncture combined with Chinese herbal medicine for the treatment of chronic nephritis: A protocol for systematic review and meta-analysis

**DOI:** 10.1097/MD.0000000000027687

**Published:** 2021-11-05

**Authors:** Xue Zheng, Shoulin Zhang, Zhilei Wang, Di Zou

**Affiliations:** a Department of Traditional Chinese Medicine, Changchun University of Chinese Medicine, 1035 Boshuo Road, Changchun, Jilin, China; b Department of Nephrology, Jilin Province Hospital of Chinese Medicine: First Affiliated Hospital to Changchun University of Chinese Medicine, 1478 Gongnong Road, Changchun, Jilin, China.

**Keywords:** acupuncture, Chinese herbal medicine, chronic nephritis, protocol, systematic review

## Abstract

**Background:**

Chronic nephritis is a common kidney disease that afflicts people worldwide. The disease has main manifestations of proteinuria, hematuria, edema, and hypertension that are associated with kidney-damaging processes that eventually lead to kidney failure. Traditional Chinese medicine involving combination treatment with herbal remedies and acupuncture has been shown clinically to alleviate chronic nephritis, although to date no systematic review of the efficacy of this combination treatment for this purpose has been reported, prompting this study. Here we conducted a systematic review and meta-analysis of published randomized clinical trials to scientific evidence and credible medical references supporting the clinical efficacy of this combination treatment when used to treat chronic nephritis.

**Methods:**

We will search the following 8 electronic Chinese and English databases: Web of Science, PubMed, Cochrane Library, Embase, China Biomedical Literature Database, China National Knowledge Infrastructure, China Scientific Journal Database, and the Wanfang database. All electronic databases will be searched from inception to October 10, 2021. All statistical analyses will be performed using Review Manager Version 5.4 provided by the Cochrane Collaboration Network.

**Results:**

The protocol for systematic review and meta-analysis will be applied to evaluate the efficacy of acupuncture combined with Chinese herbal medicine for the treatment of chronic nephritis.

**Conclusion:**

We plan to submit the results of this research to a peer-reviewed journal.

**INPLASY registration number:**

INPLASY2021100051.

## 1 Introduction

Chronic nephritis, a kidney disease that most commonly manifests as chronic glomerulonephritis, is currently viewed as a global public health problem that is increasingly attracting attention from the medical community.^[1]^ Chronic nephritis severity varies greatly, but the disease mainly manifests as hematuria, proteinuria, and hypertension or edema^[2,3]^ that eventually worsen and lead to irreversible decline of kidney function. According to statistical data, the prevalence of chronic nephritis worldwide is approximately 8% to 16%.^[4]^ The course of the disease is generally protracted and gradually progressive, although some patients experience acute exacerbations. At present, clinical treatment of this disease is challenging and prognosis is relatively poor, such that patients with chronic nephritis ultimately experience life-threatening renal failure. Current chronic nephritis treatments mainly include hormones, immunosuppressants, and symptomatic supportive therapies.^[5]^ However, long-term administration of these treatments can lead to adverse reactions, such as infection, digestive ulcers, obesity, osteoporosis, increased blood pressure, and elevated blood sugar. Based on various clinically recognized chronic nephritis symptoms, traditional Chinese medicine (TCM) syndromes associated with the disease belong to categories of “edema” and “kidney wind”.^[6]^ Importantly, TCM treatment of chronic nephritis patients can significantly alleviate clinical symptoms, improve quality of life, and promote healthier levels of 24-hour urinary total protein, hematuria, urinary microalbuminuria, and other indicators. Meanwhile, acupuncture, a green TCM therapy often used in combination with other types of TCM interventions, offers advantages of treatment effectiveness for a broad range of indications, concurrent alleviation of symptoms and root causes, a clear curative effect, simple and economical operation, safety, and few side effects.^[7]^ Notably, acupuncture combined with Chinese herbal medicine has been shown to significantly alleviate chronic nephritis,^[8,9]^ although no systematic review yet has been conducted to analyze existing clinical data to determine the safety and efficacy of acupuncture-herbal medicine combination therapy, prompting this study. Here we conducted a systematic review and meta-analysis of published randomized clinical trials (RCTs) of acupuncture-herbal medicine combination therapy to gather scientific evidence with regard to safety and efficacy of this therapy and obtain reference protocols to guide administration of this treatment in a clinical setting.

### 1.1 Protocol registration

This systematic review protocol will be implemented and strictly followed to adhere to guidelines outlined in preferred reporting items for systematic reviews and meta-analyses.^[10]^ Any changes or important amendments to the protocol will be documented in the full review. Ethical approval is not required for our study, as all analyses will be based on pooled data from previously published studies. The protocol was registered and subsequently approved online using the INPLASY website (https://inplasy.com/inplasy-2021-10-0051/). The INPLASY registration number is INPLASY2021100051.

### 1.2 Inclusion criteria

#### 1.2.1 Study design

Only randomized controlled trials (RCTs) with complete clinical data will be included and RCTs will not be restricted based on blinding, country or region or Chinese or English-language.

#### 1.2.2 Types of patients

Adult patients who have been diagnosed with chronic nephritis, without regard to region, race, sex, age, education, course of disease, or duration of treatment, will be included.

#### 1.2.3 Interventions and comparisons

All patients included in the study received routine treatment for chronic nephritis. However, patients in intervention groups additionally received acupuncture combined with Chinese herbal medicine.

#### 1.2.4 Outcomes

##### 1.2.4.1 Primary outcomes

Overall efficacy (or overall response rate).

##### 1.2.4.2 Secondary outcomes

- 24-hour urinary total protein (g/24 h).
- Serum creatinine (μmol/L).
- Blood urea nitrogen (mmol/L).
- Urine α1-microglobulin (mg/L).
- Urinary microalbuminuria (mg/L).

### 1.3 Exclusion criteria

Exclusion criteria will include the following: studies that do not meet the abovementioned inclusion criteria; animal and cell-based experiments, case reports, letters, reviews, and duplicate studies. RCTs with poor design, incomplete or incorrectly analyzed data, or studies that are unavailable publicly or obtainable from corresponding authors will also be excluded.

### 1.4 Database search strategy

We will search 8 electronic databases, including Web of Science, PubMed, Cochrane Library, Embase, China biomedical literature database, China national knowledge infrastructure, China scientific journal database, and Wanfang database (Wanfang), from date of their inception to October 10, 2021. Keywords “acupuncture”, “Chinese herbal medicine”, and “chronic nephritis” will be used to retrieve the final set of chronic nephritis-related RCTs.

### 1.5 Study selection and data extraction

Two authors (XZ and ZW) will individually review titles and abstracts to screen for relevant studies according to aforementioned predefined inclusion and exclusion criteria followed by cross-checking of results. Disagreements will be resolved through discussions with a third researcher (SZ). Studies selected will be registered in EndnoteX9.

Information associated with included studies and clinical data will be extracted and documented in Excel, including year of publication, first author, chronic nephritis patient-related data (sample size, age, sex), course of disease, interventions, treatment course, response rate, and results of laboratory examinations. For studies with unavailable full text or incomplete data, we will immediately contact the first author or corresponding authors by phone and email to try to obtain this information.

### 1.6 Risk of bias assessment

Two authors (XZ and ZW) will independently appraise the methodological quality of each trial in 6 dimensions: selection bias, performance bias, detection bias, attrition bias, reporting, and other biases. Any disagreements will be resolved through discussions between the 2 authors or consultation with a third researcher (SZ). Articles not made available with complete clinical data will be excluded after several attempts have been made to contact the authors of those studies.

### 1.7 Statistical analysis

All statistical analyses will be performed using Review Manager (RevMan) Version 5.4 (The Cochrane Collaboration, 2020. The Cochrane Collaboration St Albans House, 57-59 Haymarket, London SW1Y 4QX United Kingdom). Dichotomous variables will be expressed as odds ratio values and continuous variables will be expressed as mean difference values. Estimated values with 95% confidence interval values will be included to indicate statistical significance. All variables will be considered statistically significant at *P* < .05 unless otherwise specified. The *I*^2^ statistic and *P* value will be computed to quantify heterogeneity. A fixed-effect model will be employed when no statistical heterogeneity (*I*^2^ < 50% and *P* > .1) across studies is found; otherwise, a random-effect model will be used, with subgroup analyses conducted of sex, age, course of disease to explore potential sources of heterogeneity. Sensitivity analysis will be performed to check the robustness of conclusions and identify outliers that markedly deviated from other trials by removing 1 study at a time. Reasons for variability will be explained.

### 1.8 Analysis of subgroups or subsets

Due to potential heterogeneity that may adversely impact results of this study, after all information included in this study is collected, we could perform subgroup analysis based on sex, age, and treatment duration of all included subjects.

### 1.9 Sensitivity analysis

A sensitivity analysis will be performed to assess the robustness of the final set of results. If the results are found to be unstable, studies with a high risk of bias will be excluded.

### 1.10 Ethics and publishing

Study data will be obtained from public databases and use of such data does not require ethical approval. We will submit the final research results to a peer-reviewed journal for publication.

## 2 Discussion

Chronic nephritis is a common kidney disease^[11]^ that usually has an insidious onset, due to a lack of obvious signs and symptoms that prevents disease detection. Delayed treatment of edema associated with chronic nephritis can lead to further progression of the condition.^[2,3]^ Acupuncture, a component of traditional Chinese medicine based on insertion of needles into specific points of the human body, has a long history of use for treatment of diseases and pain relief. However, to date relatively few reported studies have focused on acupuncture combined with Chinese herbal medicine for chronic nephritis treatment, prompting us to conduct this systematic review and meta-analysis. We hope that the results of this work will provide scientific evidence and credible medical references for use in evaluating the safety and efficacy of this combination treatment for use in treating chronic nephritis patients in a clinical setting.

## Author contributions

**Conceptualization:** Xue Zheng

**Data curation:** Xue Zheng, Zhilei Wang, Di Zou

**Formal analysis:** Xue Zheng, Zhilei Wang, Di Zou, Shoulin Zhang

**Funding acquisition:** Shoulin Zhang

**Investigation:** Xue Zheng

**Methodology:** Xue Zheng, Zhilei Wang, Di Zou

**Resources:** Xue Zheng, Di Zou, Shoulin Zhang

**Software:** Xue Zheng, Zhilei Wang, Di Zou, Shoulin Zhang

**Supervision:** Xue Zheng, Shoulin Zhang

**Visualization:** Xue Zheng, Zhilei Wang, Di Zou, Shoulin Zhang

**Validation:** Xue Zheng, Zhilei Wang, Di Zou, Shoulin Zhang

**Writing – original draft:** Xue Zheng

**Writing – review & editing:** Xue Zheng, Zhilei Wang, Di Zou, Shoulin Zhang
